# Transarterial embolization of gastroduodenal peptic ulcer bleeding: a single-center study of safety and efficacy

**DOI:** 10.1007/s00423-025-03695-8

**Published:** 2025-04-02

**Authors:** Cihan Ozen, Muhanad Al-Hashimi, Mogens Tornby Stender, Ole Thorlacius-Ussing, Anders Christian Larsen

**Affiliations:** 1 Department of Surgery, Aalborg University Hospital, Hobrovej 18-22, 9100 Aalborg, Denmark; 2 Department of Radiology, Aalborg University Hospital, Aalborg, Denmark

**Keywords:** Gastroduodenal peptic ulcer, Transarterial embolization, Rebleeding, Complications, Mortality, Survival

## Abstract

**Objectives:**

To investigate the safety and efficacy of transarterial embolization (TAE) in patients with bleeding gastroduodenal peptic ulcers with an emphasis on prophylactic TAE (*p*TAE).

**Methods:**

This retrospective cohort study was conducted from 1 January 2010 to 30 June 2022. The primary outcome was rebleeding rate after TAE. Secondary outcomes were frequency and severity of complications, 30-day mortality rate, and overall survival.

**Results:**

87 patients were included. The overall rebleeding rate after TAE was 13/87 (15%). The rebleeding rate was non-significantly higher in the therapeutic TAE (*t*TAE) group (31%) when compared to the *p*TAE group (12%). Minor complications were observed in 14/87 patients (16%) and severe complications were observed in 6/87 patients (7%). The complication rate did not differ between the *t*TAE and *p*TAE groups. The 30-day overall mortality rate was 19/87 (22%). The 30-day mortality rate was non-significantly higher in the *t*TAE-group (31%) when compared to the *p*TAE group (20%). Of the 19 mortalities within 30-days, three were considered procedure-related. The overall median survival rate was 21 months (95% CI: 9.8 – 31). A non-significant higher median survival of 46.7 months (95% CI 1.2 – 74.9) was observed in the *t*TAE group when compared to 20.5 months (95% CI 7.1–29.1) in the *p*TAE group.

**Conclusion:**

TAE is safe and efficient but is associated with a high 30-day mortality rate and poor overall survival owing to a high burden of comorbidity and disease-related rather than TAE-related complications. Further studies are needed to clarify the gain and selection criteria for *p*TAE.

## Introduction

Acute gastroduodenal peptic ulcer bleeding remains one of the most common gastrointestinal emergencies and carries considerable morbidity and mortality [1]. Although modern endoscopic first-line combination therapy is associated with primary hemostasis success rates of 80–95%, 20% of patients still develop recurrent bleeding [1, 2]. During the last decades, the in-hospital mortality rate after successful endoscopic hemostasis has decreased to about 2% but increases 4- to tenfold in case of failed endoscopic hemostasis [1]. Since its introduction as salvage therapy in 1972 [3], transarterial embolization (TAE) has been widely adopted as a minimally invasive alternative to salvage surgery in case of failed endoscopic hemostasis (therapeutic TAE (*t*TAE)), but data evaluating the use of TAE as a prophylactic adjunct after successful endoscopic hemostasis (prophylactic TAE (*p*TAE)) remain limited. *p*TAE has been considered particularly suitable in lesions with a high risk of rebleeding and in patients who are poor candidates for surgery. The selection criteria and the value of pTAE is, however, a matter of an ongoing debate [4–11]. TAE is typically performed by percutaneous transfemoral catheterization with selective cannulation of the celiac axis and superior mesenteric artery to identify the site of bleeding and delineate vascular anatomy. Embolization materials – e.g. coils – are then deployed through a coaxially inserted microcatheter by super-selective cannulation of the bleeding or likely offending artery [12]. Although TAE among many authors has been accepted as safe and effective, the rate of post-embolic bleeding is reported to be up to 25% and the mortality rate within 30 days as high as 15–26% [4, 5]. Besides well-established risk factors for ulcer bleeding including *Helicobacter pylori* infection, nonsteroid anti-inflammatory drugs (NSAIDs), and acetylsalicylates together with platelet inhibitors and anticoagulant drugs, great emphasis has been placed to identify risk factors for rebleeding and mortality after therapy, to optimize treatment algorithms [4, 13–15]. Among others, patient-related risk factors for rebleeding and adverse outcomes in upper gastrointestinal bleeding include the Rockall score, the Charlson comorbidity index (CCI), and The American Society of Anesthesiologists (ASA) score [16–20]. The Rockall score combines age, hemodynamics, comorbidity, nature of the bleeding lesion type, and stigmata of recent bleeding (Forrest classification) into a risk estimate for an adverse outcome [1, 13, 21–23]. The Rockall score has been proposed as a valuable tool in selection for TAE, but data are limited [4, 24]. In addition to patient-related risk factors, procedure-related risk factors have also been identified: e.g. use of coils alone versus a combination of embolization materials, deviation from a standardized embolization procedure, and more than one endoscopy before embolization [4, 12]. Procedure-related complications of major concern include embolization-related ischemia in the involved vascular territory, ulcer formation, and stenosis [5]. The purpose of this study was to investigate the safety and efficacy of TAE in gastroduodenal peptic ulcer bleeding in our institution with an emphasis on pTAE and to evaluate possible risk factors for rebleeding and mortality after TAE.

## Material and methods

A retrospective, single-center cohort study was conducted from 1 January 2010 to 30 June 2022 at the Department of Gastrointestinal Surgery and the Department of Radiology, Aalborg University Hospital Denmark. All patients with non-malignant gastroduodenal peptic ulcer-bleeding who underwent TAE were identified through the Patient Administration System, which is the health care system and electronic medical record in Denmark, using the ICD-10 examination codes UXAD 40 (coeliacography) and UXAD 45 (superior mesenteric artery angiography) and were considered for inclusion in the study. Patients who were younger than 18 years, underwent primary surgical treatment, with non-gastroduodenal, non-peptic bleedings (e.g. liver, spleen, pancreas, or other gastrointestinal tract bleeding), with no index endoscopy performed before TAE or who were inaccessible for TAE, were excluded. Demographic and clinical data were obtained by retrospective chart review. Information on the use of proton pump inhibitors (PPI), non-steroidal anti-inflammatory drugs (NSAID), selective serotonin reuptake inhibitors (SSRI), glucocorticoids, platelet inhibitors (i.e., clopidogrel, ticagrelor), non-vitamin K oral anticoagulants (“NOACs”, i.e. dabigatran, apixaban, rivaroxaban) and vitamin K antagonists (VKA) was documented and qualified during the admission of the patient. Clinical data collected at hospital admission included age, sex, blood pressure, CCI, hemoglobin level (mmol/L), and results of endoscopies including Forrest and Rockall scores [21, 22]. Blood transfusions before and after TAE were registered together with data on length of hospital stay, morbidity, and mortality. Data were collected using the REDcap electronic data capture tool hosted at Aalborg University Hospital REDcap.

The primary outcome was the rebleeding rate after TAE. Secondary outcomes were frequency and severity of complications, 30-day mortality rate, and overall survival.

### Initial treatment and TAE procedure

All patients were treated according to the Danish Society of Gastroenterology and Hepatology guidelines on bleeding gastroduodenal ulcers [25]. At arrival, patients were resuscitated according to the ABCDE principles. Patients with normal hemodynamics at arrival were observed in a specialized section with specially trained staff. At arrival, ASA, NSAID, Clopidogrel, vitamin K antagonist, and SSRI treatment were stopped. In continuously hemodynamically unstable patients, urgent endoscopy was performed on vital indication. If bleeding was suspected in otherwise hemodynamically stable patients, endoscopy was performed within 12–24 h after admission. In hemodynamically stable patients without suspicion of severe bleeding, endoscopy was performed within 24–48 h. Forrest I-IIb ulcers were treated endoscopically with injection of 13–25 ml of adrenaline saline as the first modality to get an overview of the bleeding site. After adrenaline saline injection, a secondary treatment modality was supplemented in the form of contact thermal probe, argon plasma coagulation, or hemoclip application. At the completion of the endoscopy, a Rockall score was assigned. If primary hemostasis could not be achieved endoscopically, TAE was performed without delay. Surgery was performed If TAE was not possible. In high-risk patients with successful endoscopic hemostasis, a prophylactic TAE was considered on an individual basis at the endoscopist’s discretion. Low-risk ulcers (Forrest IIc-III) were treated with standard oral PPI. High-risk ulcers (Forrest I-IIb) were treated with PPI in the form of 80 mg IV bolus, followed by 8 mg/h continuous infusion for 72 h. Any Helicobacter pylori infection was eradicated.

**Fig. 1:**
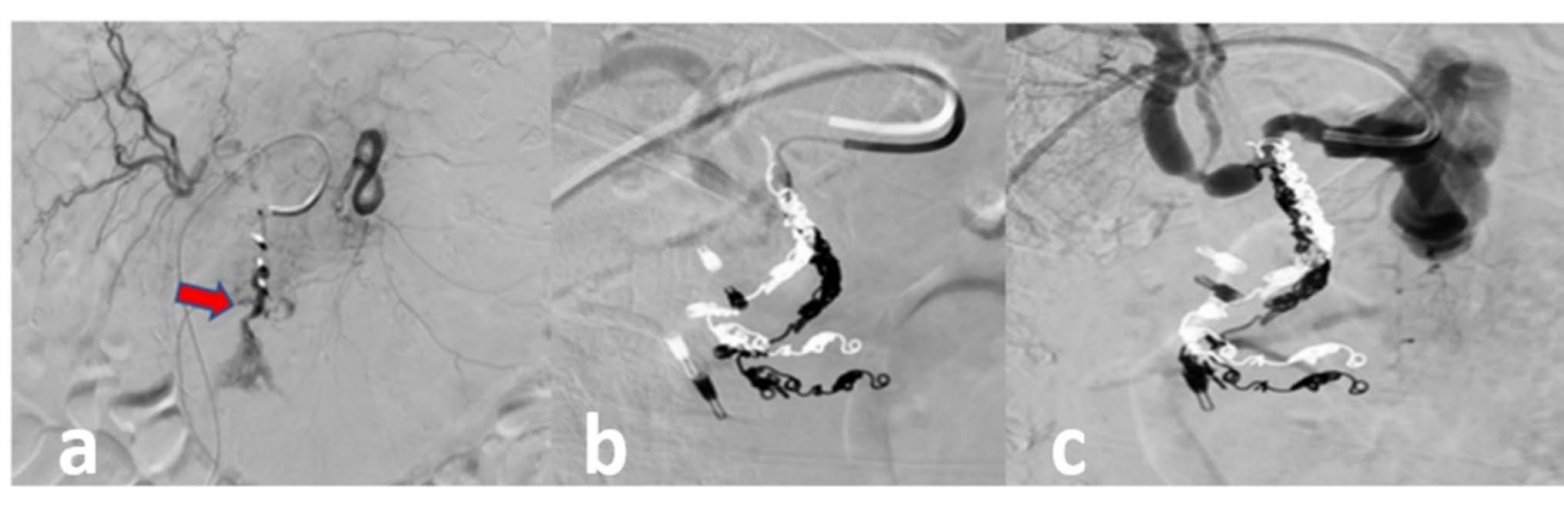
Illustration of active bleeding from the gastroduodenal artery (red arrow), before (a) and after transarterial embolization with coils (b + c)

All TAE procedures were performed with transfemoral catheterization. A mesenteric angiogram was done with selective cannulation of the celiac axis and superior mesenteric artery to identify the site of contrast extravasation and delineate vascular anatomy. If contrast extravasation was apparent, a microcatheter was inserted coaxially for super-selective cannulation of the bleeding artery. Micro coils were deposited to the bleeding artery in a distal to proximal manner until extravasation ceased together with complete occlusion of the bleeding vessel (Fig. 1). In the absence of active extravasation, the deposition of coils was guided by hemoclips placed during endoscopy. Data on TAE-related technical details (angiograms, catheter types, embolization materials etc.) were extracted from the local prospective angiography database at Dept. of Radiology, Aalborg University Hospital. The term “prophylactic TAE” (*p*TAE) refers to TAE to prevent rebleeding after successful endoscopic hemostasis. The term “therapeutic TAE” (*t*TAE) refers to TAE-targeted selective embolization in patients with failed endoscopic hemostasis.

### Rebleeding

Rebleeding was defined as redevelopment of symptoms of peptic ulcer bleeding (hematemesis and/or melena) within seven days of the latest hemostatic procedure associated with a significant decline in hemoglobin level or confirmed by endoscopy, surgery or imaging [20]. At the first rebleeding after index endoscopy, endoscopic treatment was repeated if technically feasible. In the case of repeated bleeding repeated therapeutic endoscopy, TAE, or surgery was considered on an individual basis at the treating physician’s discretion.

### Statistics

Categorical variables are presented as frequencies with percentages. Continuous variables are presented as means with 95% confidence intervals (95% CI), or medians with ranges. Fischer’s exact test was used to test for differences in proportions. The unpaired T-test was used for comparison of means for continuous normally distributed variables, and the Mann–Whitney U test was used for comparison of non-normally distributed variables. Kaplan–Meier survival analysis was performed, and survival curves were constructed according to TAE type and ASA score, respectively. The follow-up time was calculated from the date of index hospital admission. Censoring occurred at the end of the study (May 8th, 2023) or death. No patients were lost to follow-up. The Cox proportional hazard model was used to analyze the prognostic effect of variables on the overall survival. Exposure variables were defined as: age (< 60, 60–75, > 75 years), gender (male/female), location of ulcer (stomach/duodenum), Forrest Classification (Ia-b, IIa-c, III), ASA score (II-III, IV), CCI (< 4, 4–8, > 8), Rockall score (≤ 7/ > 7), NSAID (yes/no), PPI (yes/no), NOAC (yes/no), thrombocyte inhibitors (yes/no), VKA (yes/no), systolic blood pressure at arrival (mmHg) (< 75/75–100/ > 100), and hemoglobin level at arrival (mmol/L) (< 4.3/4.3–5.6/ > 5.6). The proportional hazards assumption was fulfilled by the data. A two-sided *p-*value of 0.05 was considered significant. Data were analyzed and graphs were created using STATA 15.1 (Stata Corp LLC, College Station, TX). The study adhered to the STROBE checklist (https://www.strobe-statement.org/checklists/).

### Ethics

The study was reported to The North Denmark Region Committee on Health Research Ethics with approval on 4 July 2022. Data retrieval from medical records was approved by the local research board at Aalborg University Hospital on 9 May 2022 with ID number F2022-071.

## Results

**Fig. 2:**
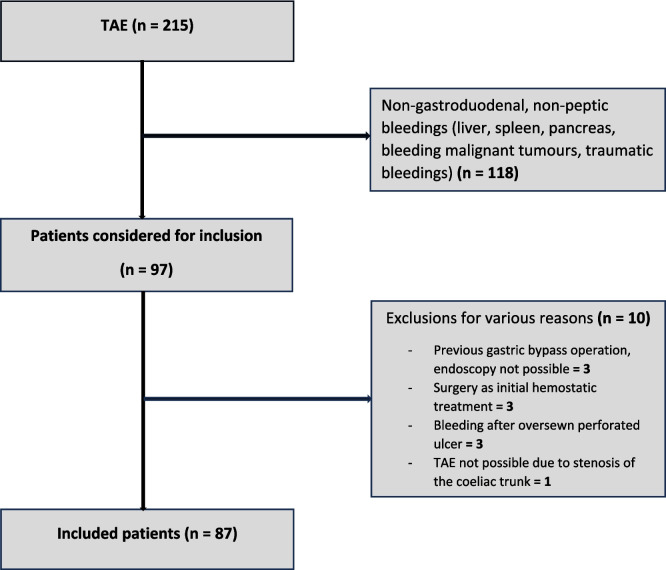
Flowchart of patient selection. Abbreviation: TAE, transarterial embolization

**Fig. 3:**
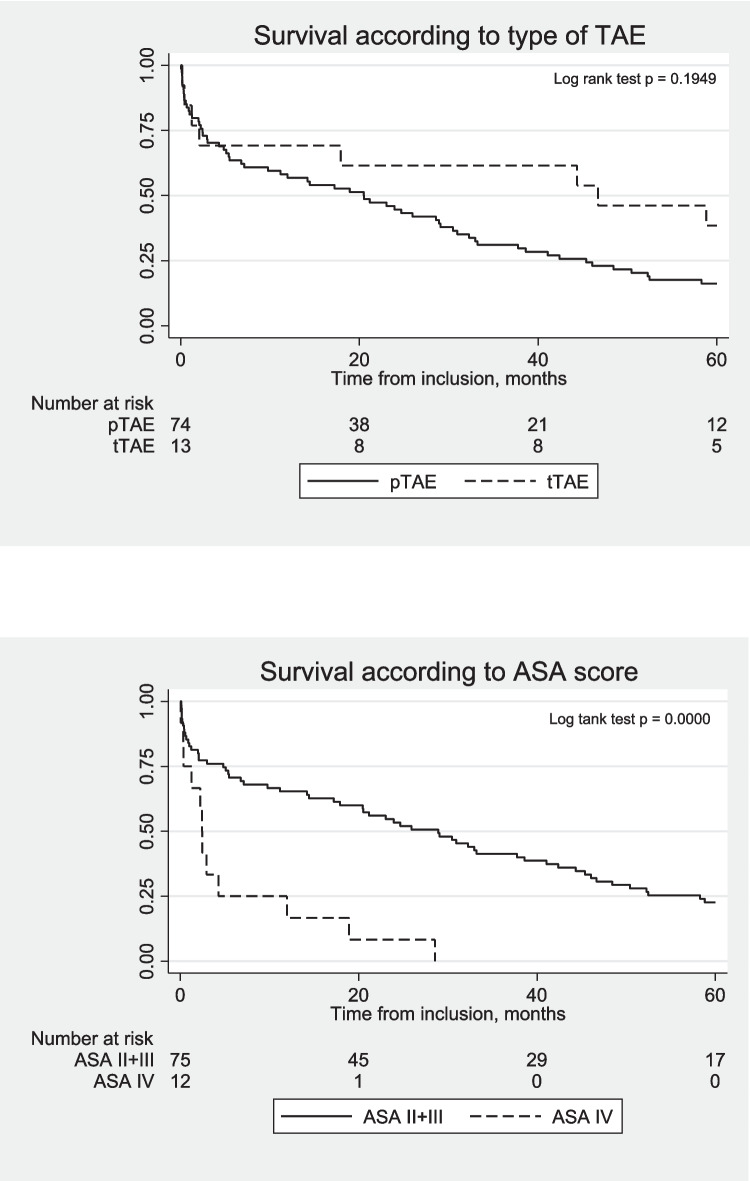
Kaplan–Meier cumulative survival estimates according to type of TAE and ASA score, respectively. Abbreviations: ASA, American Society of Anesthesiologists; pTAE, prophylactic transarterial embolization; tTAE, therapeutic transarterial embolization

A total of 215 patients receiving TAE were identified between 2010 and 2022. One hundred-eighteen patients were excluded due to non-gastroduodenal, non-peptic bleedings (liver, spleen, pancreas, bleeding malignant tumors, or traumatic bleedings). Of the remaining 97 patients, 10 were excluded for various reasons (three patients had a previous gastric bypass operation and endoscopy treatment could not be applied, three patients underwent surgery as the initial hemostatic treatment with recurrent bleeding, three patients had oversewn a perforated ulcer with recurrent bleeding at the sutured site, and TAE could not be performed in one patient due to stenosis of the coeliac trunk), leaving 87 patients for analyses (Fig. 2*)*. Demographic and clinical data are depicted in Table 1*.* The total follow-up time was 2806 months. The median follow-up time was 32.3 months (95% CI 21.1 – 142). Most patients were males, had a duodenal bleeding site, or were previous or current tobacco smokers (Table 1*).* The distribution of *H. pylori* status among the TAE groups reached statistical significance, but data on *H. pylori* status were missing in 53% of the patients. Thus, data on *H. pylori* status were omitted from further analyses. The overall rebleeding rate after TAE was 15% with the observation of a non-significant higher rebleeding rate of 31% in the *t*TAE group when compared to 12% in the *p*TAE group (Table 1*)*. Except for the utilization of a significantly higher number of coils among rebleeders when compared to non-rebleeders, no differences in the distribution of possible risk factors of rebleeding among rebleeders and non-rebleeders were observed (Table 2*).* The overall 30-day mortality rate was 19%. The 30-day mortality rate was higher in the *t*TAE group (31%) when compared to the *p*TAE group (20%), but did not reach statistical significance (Table 1*, *Fig. 3*).* 20 patients (23%) experienced procedure-related complications, of which the majority were minor. Major procedure-related complications occurred in six patients (7%), of whom three died (3%) (Table 1*).* The overall medical and surgical 30-day morbidity, according to the Clavien-Dindo scoring system, was 53%, reaching a severity grade III or IV in 23% of the patients. The complication rate did not differ between the *t*TAE and *p*TAE groups. The overall median survival was 21.2 months (95% CI 9.8–30.9) with the observation of a non-significant higher median survival of 46.7 months (95% CI 1.2 – 74.9) in the *t*TAE group, when compared to 20.5 months (95% CI 7.1–29.1) in the *p*TAE group (Table 1*, *Fig. 3). An ASA score of IV was identified as a strong predictor of mortality after TAE (Table 3*, *Fig. 3*).* No other risk factors for mortality were identified.

**Table 1:** Characteristics and outcomes of the study patients

	**TAE, overall** (*n* = 87)	***t*TAE** (*n* = 13)	***p*TAE** (*n* = 74)	**P-value**
**Age,** median (range)	77 (48–95)	79 (57–92)	77 (48–95)	*n.s*
**Gender**, *n*(%)				
male/female	53/34 (61/39)	5/8 (38/62)	48/26 (65/35)	*n.s*
**Ulcer location**, *n*(%)				
stomach/duodenum	3/84 (3/97)	1/12 (8/92)	2/72 (3/97)	*n.s*
**Forrest Classification**, *n*(%)				
Ia – Ib	48 (55)	8 (62)	40 (54)	
IIa – Iic	26 (30)	3 (23)	23 (31)	
III	13 (15)	2 (15)	11 (15)	*n.s*
**Rockall Score**, *n*(%)				
≤ 7	46 (53)	6 (46)	40 (54)	
> 7	41 (47)	7 (54)	34 (46)	*n.s*
**H. pylori**, *n*(%)				
Positive	10 (11)	3 (23)	7 (9)	
Negative	31 (36)	1 (8)	30 (41)	
Missing data	46 (53)	9 (69)	37 (50)	*0.03*
**CCI**, mean (95% CI)	5.5 (5.0–6.0)	5.3 (3.6–7.0)	5.5 (5.0–6.0)	*n.s*
**ASA Score**, *n*(%)				
II-III	75 (86)	13 (100)	62 (84)	
IV	12 (14)	-	12 (16)	*n.s*
**NSAID**, *n*(%)	16 (18)	3 (23)	13 (18)	*n.s*
**PPI**, *n*(%)	16 (18)	1 (8)	15 (20)	*n.s*
**NOAC**, *n*(%)	3 (3)	-	3 (4)	*n.s*
**Thrombocyte inhibitors**, *n*(%)	3 (3)	-	3 (4)	*n.s*
**VKA**, *n*(%)	5 (6)	2 (15)	3 (4)	*n.s*
**Smoking status**, *n*(%)				
Never	30 (34)	3 (23)	27 (36)	
Current	36 (41)	7 (54)	29 (39)	
Previously	19 (22)	3 (23)	16 (22)	
Missing data	2 (2)	-	2 (3)	*n.s*
**Alcohol abuse**, *n*(%)	26 (30)	6 (46)	20 (27)	*n.s*
**Systolic BP < 100 mmHg at arrival**, *n*(%)	41 (47)	5 (39)	36 (49)	*n.s*
**Hemoglobin level at arrival** (mmol/L), *n*(%)				
> 5.6	18 (21)	2 (15)	16 (22)	
4.3–5.6	30 (34)	4 (31)	26 (35)	
< 4.3	39 (45)	7 (54)	32 (43)	*n.s*
**Number of coils**, *n*(%)				
< 12	30 (34)	5 (38)	25 (34)	
13–25	42 (48)	7 (54)	35 (47)	
> 25	14 (16)	1 (8)	13 (18)	
Unknown	1 (1)	**-**	1 (1)	*n.s*
**Outcomes**				
**Rebleeding after TAE**, *n*(%)	13 (15)	4 (31)	9 (12)	*n.s*
Re-intervention				
- Endoscopy - Surgery - Observation	4 (5) 5 (6) 4 (5)	1 (8) 3 (23) -	3 (4) 2 (3) 4 (5)	
**30-day mortality after TAE**, n(%)	19 (22)	4 (31)	15 (20)	*n.s*
**Median survival**, months (95% CI)	21.2 (9.8 – 30.9)	46.7 (1.2 – 74.9)	20.5 (7.1 – 29.1)	*n.s*
**30-day procedure-related complications**, n(%)	20 (23)	3 (23)	17 (23)	
**Major**	6 (7)	1 (8)	5 (7)	
Death	3 (3)	1 (8)	2 (3)	
Coil displacement with bowel ischemia	1(1)	-	1 (1)	
Coil displacement with splenic infarction	1(1)	-	1 (1)	
Bowel ischemia without perforation	3(5)	-	3 (4)	
Bowel ischemia with perforation	1(8)	1 (8)	-	
**Minor**	14 (16)	2 (15)	12 (16)	
Coil displacement without ischemia	11 (13)	1 (8)	10 (14)	
Other (inguinal hematoma etc.)	3 (3)	1(8)	2 (3)	
**30-day overall morbidity (Clavien-Dindo)** [29], n(%)	46 (53)	9 (69)	37 (50)	
Grade I	7 (8)	1 (8)	6 (8)	
Grade II	-	-	-	
Grade III	8 (9)	3 (23)	5 (7)	
Grade IV	12 (14)	1 (8)	11 (15)	
Grade V	19 (22)	4(31)	15 (20)	*n.s*
**Length of hospital stay** (days), median (range)	8 (3–48)	8 (5– 45)	8 (3 – 48)	*n.s*

Abbreviations*: ASA* American Society of Anesthesiologists, *BP* Blood pressure, *CCI* Charlson Comorbidity Index, *CI* Confidence interval, *NOAC* Non-vitamin K oral anticoagulant, *NSAID* Non-steroid anti-inflammatory drugs, *PPI* Proton pump inhibitors,*VKA* vitamin K antagonists

**Table 2:** Characteristics of rebleeders versus non-rebleeders

	**TAE, overall** (*n* = 87)	**Rebleeding after TAE** (*n* = 13)	**No rebleeding after TAE** (*n* = 74)	**P-value**
**Age**, median (range)	77 (48–95)	79 (53–85)	76 (48–95)	*n.s*
**Gender**, *n*(%)				
Male/female	53/34 (61/39)	8/5 (62/38)	45/29 (61/39)	*n.s*
**Ulcer location**, *n*(%)				
stomach/duodenum	3/84 (3/97)	1/12 (8/92)	2/72 (3/97)	*n.s*
**Forrest Classification**, *n*(%)				
Ia – Ib	48 (55)	7 (54)	41 (55)	
IIa – Iic	26 (30)	5 (38)	21 (28)	
III	13 (15)	1 (8)	12 (16)	*n.s*
**Rockall score**, *n*(%)				
≤ 7	46 (53)	7 (54)	39 (53)	
> 7	41 (47)	6 (46)	35 (47)	*n.s*
**ASA Score**, *n*(%)				
II-III	75 (86)	12 (92)	63 (85)	
IV	12 (14)	1 (8)	11 (15)	*n.s*
**CCI**, mean (95% CI)	5.5 (5.0–6.0)	5.7 (4.0–7.4)	5.4 (4.9–5.9)	*n.s*
**NSAID**, *n*(%)	16 (18)	4 (31)	12 (16)	*n.s*
**PPI**, n(%)	16 (18)	1 (8)	15 (20)	*n.s*
**NOAC**, *n*(%)	3 (3)	0 (0)	3 (4)	*n.s*
**Thrombocyte inhibitors**, *n*(%)	3 (3)	0 (0)	3 (4)	*n.s*
**VKA**, *n*(%)	5 (6)	2 (15)	3 (4)	*n.s*
**Systolic BP < 100 mmHg at arrival**, *n*(%)	41 (47)	5 (38)	36 (49)	*n.s*
**Hemoglobin level at arrival** (mmol/L), *n*(%)	18 (21)	1 (8)	17 (23)	
> 5.6	30 (34)	5 (38)	25 (34)	
4.3–5.6	39 (45)	7 (54)	32 (43)	*n.s*
< 4.3				
**Number of coils**, n(%)				
< 12	30 (34)	1 (8)	29 (39)	
13–25	42 (48)	10 (77)	32 (43)	
> 25	14 (16)	2 (15)	12 (16)	
Unknown	1 (1)	-	1 (1)	*0.040*

Abbreviations:* ASA* American Society of Anesthesiologists, *BP* Blood pressure, *CCI* Charlson Comorbidity Index, *CI* Confidence interval, *NOAC* Non-vitamin K oral anticoagulant, *NSAID* Non-steroid anti-inflammatory drugs, *PPI* Proton pump inhibitors, *VKA* Vitamin K antagonists

**Table 3:** Risk factors for overall mortality. Uni- and multivariate analyses

	**Univariate analysis** Hazard ratio (95% CI)	***P*-value**	**Multivariate analysis** Hazard ratio (95% CI)	***P*-value**
**Age**				
< 60	1.00		1.00	
60–69	1.51 (0.66 – 3.44)		1.68 (0.72 – 3.90)	
> 70	1.30 (0.65 – 2.57)	*n.s*	1.23 (0.55 – 2.77)	*n.s*
**Gender**				
Male	1.00		1.00	
Female	1.11 (0.71 – 1.72)	*n.s*	1.02 (0.59 – 1.76)	*n.s*
**Ulcer location**				
Stomach	1.00		1.00	
Duodenum	3.99 (0.96 – 16.54)	*0.056*	6.01 (1.24 – 29.28)	*0.026*
**Forrest Classification**				
III	1.00		1.00	
IIa – Iic	0.91 (0.47 – 1.79)		0.77 (0.36 – 1.65)	
Ia – Ib	0.75 (0.49 – 1.68)	*n.s*	1.17 (0.51 – 2.68)	*n.s*
**Rockall score**				
≤ 7	1.00		1.00	
> 7	1.04 (0.68–1.59)	*n.s*	0.89 (0.54 – 1.49)	*n.s*
**ASA Score**				
II-III	1.00		1.00	
IV	3.79 (1.93–7.42)	*0.000*	3.43 (1.52 – 7.71)	*0.003*
**CCI**				
< 4	1.00		1.00	
4–8	1.29 (0.82 – 2.03)		1.57 (0.85 – 2.88)	
> 8	2.04 (0.89 – 4.65)	*n.s*	1.46 (0.49 – 4.34)	*n.s*
**TAE type**				
*p*TAE	1.00		1.00	
*t*TAE	0.68 (0.37–1.23)	*n.s*	0.72 (0.37–1.37)	*n.s*

Abbreviations: *ASA* American Society of Anesthesiologists, *CCI* Charlson Comorbidity Index, *CI* Confidence interval, *TAE* Transarterial embolization, *pTAE* Prophylactic transarterial embolization, *tTAE* therapeutic transarterial embolization

## Discussion

In the present study, investigating the safety and efficacy of TAE among 87 patients with gastroduodenal peptic ulcer bleeding, we were able to achieve permanent hemostasis in 85% of the patients after TAE, corresponding to a rebleeding rate of 15%. Only six percent of the patients needed salvage hemostatic surgery. The overall medical and surgical morbidity rate was high (53%), but the rate of severe procedure-related complications per se was low (7%), translating into a procedure-related mortality rate of only three percent. However, we observed a very high overall 30-day mortality rate of 22%, and an overall median survival of only 21.2 months, mainly attributable to the high-risk character of these older patients, carrying a high burden of co-morbidities and complications in general, unrelated to the TAE procedure per se. We observed an extremely poor survival in ASA IV patients, and, not surprisingly, the ASA score was identified as a strong predictor of mortality. We failed to identify any other risk factors of mortality, but a trend of higher mortality was observed among patients in the *p*TAE group and with duodenal bleeding sites.

Our estimates of overall rebleeding rate, complication rate, and mortality are in concordance with recent previous studies [4, 18]. The slightly higher rate of rebleeding (31%) in the *t*TAE group, when compared to the *p*TAE group (12%), is in line with a newly published study from 2023 of McGraw et al., who found rebleeding in 69 out of 269 patients (25%) who underwent acute therapeutic TAE [5]. The rebleeding rate is also comparable to the reported rate in a metanalysis of 11 mainly retrospective studies. Inhere, rebleeding occurred in 116 out of 327 patients (35%) who underwent *t*TAE [26].

In contrast to *t*TAE, the role of *p*TAE is yet to be established. In a meta-analysis by Chang et al. from 2020, including five randomized controlled trials (RCTs), it was demonstrated that the addition of TAE after successful endoscopic hemostasis improved outcomes in terms of rebleeding, reintervention, and mortality rates, when compared to endoscopic therapy without the addition of TAE [6]. However, the meta-analysis included an RCT from 2014 by Laursen et al., who did not demonstrate an improvement in outcomes in their *p*TAE group. The study lacked, however, statistical power and did not meet the target sample size to be included in each arm [6, 11]. Similarly, an RCT by Lau et al. failed to show any difference in outcomes, except reduced rebleeding in a subgroup with ulcers ≥ 15 mm [6, 7]. In a retrospective cohort study of patients receiving *p*TAE, Zetner et al. observed a higher risk of rebleeding, when compared to other studies, but the adverse event rate and mortality rate were similar [4]. In the present study, the rebleeding rate of 12% in the pTAE group is competitive with the reported outcomes in the Chang meta-analysis, which reported rebleeding rates from 3.4% to 11% [6]. In addition, the rate of major complications and 30-day mortality rate in our *p*TAE group was favorably compared with the *t*TAE group. Thus, we have reason to believe, that *p*TAE is safe and effective in our setting, although no standardized selection criteria for *p*TAE were utilized.

Surprisingly, neither the Forrest Classification, Rockall score, nor CCI predicted mortality after TAE in the present study. Trends were, however, observed in the multivariate analysis. We speculate that the lack of predictive power is mainly attributable to the small sample size. Thus, larger prospective studies are needed including a validation of the relevant cut-off value for the Rockall score in the present clinical setting [4, 24].

Similar to other studies, we found that the occurrence of rebleeding was associated with a higher number of coils used during the TAE procedure. We speculate that the number of coils needed is a surrogate of the size of the bleeding lesion. Our finding underscores that a successful TAE treatment might be highly dependable on the technique and utensils utilized. The main part of the reported studies, inclusive of the present study, is retrospective and composed of a mix of different utensils and embolization materials. Some studies report on the use of foam or gels as embolization materials, and some studies advocate for the use of a combination of embolization materials, which makes it difficult to interpret the results and compare studies [6, 27, 28].

Strengths of this study include a complete and highly detailed follow-up of all patients due to exhaustive electronic medical records providing electronic access to the local prospective angiography database, allowing for re-evaluation of examinations and extraction of data regarding procedure-related details (e.g. angiograms, catheter types, embolization materials, etc.)

The study carries several limitations. First, the retrospective study design did not allow for any control of exposure or outcome assessments. Second, the rather small sample size might have blurred any true association between exposure variables and outcomes due to a lack of statistical power. Especially, the evaluation of possible risk factors of rebleeding was severely compromised by the very few rebleeding events (thirteen), which did not allow for logistic regression analyses. Third, the lack of an appropriate control group makes our outcome estimates hard to interpret. Furthermore, treatments and techniques might have improved during the 12-year inclusion period. However, all TAEs have been performed by the same three interventional radiologists with no major changes in techniques, and the endoscopic, surgical, and medical ulcer treatments have not changed significantly during the inclusion period.

In conclusion, TAE in patients with gastroduodenal peptic ulcer bleeding is safe and efficient but is associated with a high 30-day mortality rate and poor overall survival, primarily owing to a high burden of comorbidity and disease-related rather than TAE-related complications. The study did not provide evidence against pTAE, but further randomized controlled trials are needed to clarify the gain and selection criteria for pTAE.

## Data Availability

Data sets generated during the current study are available from the corresponding author on reasonable request.
